# PD203 Comparison Of Health Technology Assessment Methodologies Across Australia, Canada, New Zealand And The United Kingdom: Implications For Future Collaboration

**DOI:** 10.1017/S0266462324004252

**Published:** 2025-01-07

**Authors:** Nadine Henderson, Claud Theakston, Simon Brassel, Martina Garau, Rachel Allen, Nathalie Largeron, Kinga Malottki, Yuti Patel, Kirsten Garces, Megan Coombes, Vanessa Xavier

## Abstract

**Introduction:**

In 2022, a group of health technology assessment (HTA) bodies from Australia, Canada, and the UK announced a collaboration to identify solutions to common challenges. This collaboration was later expanded to include agencies from New Zealand and Quebec, Canada. Since one possible activity of the consortium is joint assessments, we compared the methodologies of the agencies on 11 topics to assess the feasibility of this.

**Methods:**

We reviewed the methodological guidelines of the Canadian Agency for Drugs and Technologies in Health (CADTH), L’Institut national d’excellence en santé et services sociaux (INESSS), the National Institute for Health and Care Excellence (NICE), the Pharmaceutical Benefits Advisory Committee (PBAC), the Pharmaceutical Management Agency (Pharmac), and the Scottish Medicines Consortium (SMC). The topics considered were real-world evidence, consideration of health effects, economic reference case, survival analysis, surrogate endpoints, patient involvement, uncertainty, orphan pathways, clinical evidence requirements, carer perspective, and decision modifiers. We analyzed the level of alignment across the collaborating agencies using information from the guidelines, supplemented by published literature where necessary.

**Results:**

Three topics exhibited high alignment: consideration of health effects, clinical evidence requirements and surrogate endpoints. The topics of orphan pathways and carer perspective had low alignment. The remaining topics had moderate alignment. Regarding orphan pathways, NICE and the SMC had separate processes for ultra-orphan drugs, CADTH and INESSS implicitly consider rarity, and PBAC and Pharmac do not appear to consider rarity. Since carer perspective is not commonly accepted in HTA, NICE was the only agency with relevant guidance on this topic. INESSS required the societal perspective as standard, while the PBAC and Pharmac explicitly excluded it. CADTH may consider carer perspective in some circumstances, whereas the SMC guidance was ambiguous.

**Conclusions:**

While there is good alignment on most topics, there are several areas where agencies would need to resolve divergences in preferred methodology if joint assessments are going to be carried out in the future. All relevant stakeholders should be part of this process, including patient groups and industry.

